# Current Controversy on Platelets and Patent Ductus Arteriosus Closure in Preterm Infants

**DOI:** 10.3389/fped.2021.612242

**Published:** 2021-02-25

**Authors:** Hannes Sallmon, Natalie Timme, Begüm Atasay, Ömer Erdeve, Georg Hansmann, Yogen Singh, Sven C. Weber, Elaine L. Shelton

**Affiliations:** ^1^Department of Pediatric Cardiology, Charité University Medical Center, Berlin, Germany; ^2^Department of Congenital Heart Disease/Pediatric Cardiology, Deutsches Herzzentrum Berlin (DHZB), Berlin, Germany; ^3^Division of Neonatology, Department of Pediatrics, Ankara University School of Medicine, Ankara, Turkey; ^4^Department of Pediatric Cardiology and Intensive Care Medicine, Medizinische Hochschule Hannover, Hanover, Germany; ^5^Department of Neonatology and Pediatric Cardiology, Cambridge University Hospitals, Cambridge, United Kingdom; ^6^University of Cambridge School of Clinical Medicine, Cambridge, United Kingdom; ^7^Department of Pediatrics, Vanderbilt University Medical Center, Nashville, TN, United States; ^8^Department of Pharmacology, Vanderbilt University Medical Center, Nashville, TN, United States

**Keywords:** platelets, thrombocytopenia, very low birth weight, patent ductus arteriosus, hemodynamics

## Abstract

Platelets are critically involved in murine patent ductus arteriosus (PDA) closure. To date, the clinical significance of these findings in human preterm infants with PDA is still controversial. We discuss the available study data on the role of platelets for PDA closure in preterm infants: Several mostly retrospective studies have yielded conflicting results on whether thrombocytopenia contributes to failed spontaneous ductal closure. The same applies to investigations on the role of thrombocytopenia as a risk factor for unsuccessful ductus arteriosus closure by pharmacological treatment with cyclooxygenase inhibitors. Nonetheless, recent meta-analyses have concluded that thrombocytopenia constitutes an independent risk factor for both failed spontaneous and pharmacological PDA closure in preterm infants. However, the available investigations differ in regard to patient characteristics, diagnostic strategies, and treatment protocols. Several studies suggest that impaired platelet function rather than platelet number is critically involved in failure of ductus arteriosus closure in the preterm infant. A recent randomized-controlled trial on platelet transfusions in preterm infants with PDA failed to show any benefit for liberal vs. restrictive transfusion thresholds on PDA closure rates. Importantly, liberal transfusions were associated with an increased rate of intraventricular hemorrhage, and thus should be avoided. In conclusion, the available evidence suggests that thrombocytopenia and platelet dysfunction contribute to failure of spontaneous and pharmacological PDA closure in preterm infants. However, these platelet effects on PDA seem to be of only moderate clinical significance. Furthermore, platelet transfusions in thrombocytopenic preterm infants in order to facilitate PDA closure appear to cause more harm than good.

## Introduction

After birth, a persistently patent ductus arteriosus (PDA) is usually associated with left-to-right shunt; and subsequently, variable degrees of pulmonary overcirculation, systemic hypotension, and malperfusion (1). In preterm infants, the consequences of a hemodynamically significant PDA (hsPDA) are frequently associated with several severe cardiopulmonary complications such as left ventricular volume overload, pulmonary edema, impairment of lung compliance, and abdominal-renal malperfusion due to the ductal steal (1–3). Thus, understanding the mechanisms that contribute to ductus arteriosus (DA) closure is of pivotal importance in order to provide tailored care to at-risk neonates (4, 5).

In 2010, Echtler et al. (6) reported that platelets contribute to ductal closure and subsequent vascular remodeling in mice. By means of intravital microscopy, they showed that platelets are recruited to the luminal aspect of the ductal endothelium within minutes after birth and that formation of a platelet-plug within the DA contributes to ductal remodeling. In addition, by using two murine models of genetic disruption of platelet biogenesis and function (Nfe^−/−^ and Itga2b^−/−^ mice), the authors demonstrated that platelet dysfunction was associated with hsPDA (as assessed by a quantification of pulmonary blood flow using radiolabeled microspheres). Similar results were observed when an antibody directed against platelet collagen receptor GPVI was administered, thus indicating that both GPVI and GPIIb/IIIa contribute to platelet-triggered DA closure. In addition, the authors showed that administration of cyclooxygenase inhibitors (ibuprofen or indomethacin) was not sufficient to compensate for impaired platelet function in their murine model (6). Of note, mouse ductuses are remarkably similar to human ductuses, but they lack prominent intimal cushions, which protrude into the vessel lumen and contribute to vessel closure and permanent remodeling. Therefore, platelets may play a larger role in mouse vs. human duct occlusion due to the lack of intimal cushions.

Taken together, the experimental results above suggest that platelets play a pivotal role in ductus arteriosus closure. However, the clinical significance of these findings in human preterm infants is still controversial and has not been confirmed *in vivo* (7). Here, we discuss the available data on the current controversy on platelets and PDA and their clinical implications in preterm infants.

## The Effects of Thrombocytopenia and Platelet Transfusions on Spontaneous and Pharmacological Ductus Arteriosus Closure

Low platelet counts are frequently observed among preterm infants (8, 9) and the role of platelets in spontaneous or pharmacological PDA closure has been explored by several groups yielding conflicting results. In addition to their animal results, Echtler et al. (6) also reported on a small cohort of 123 preterm infants, in which low platelet counts within the first 24 h after birth were associated with a higher incidence of PDA. However, another small study (*n* = 211) failed to confirm these results in a Japanese cohort of preterm infants (10). Similarly, Shah et al. could not find any association between low platelet counts during the 1st week of life and incidence of PDA, but they observed a decreased incidence of PDA when platelet counts were consistently > 230/nL. Of note, all infants in this study received prophylactic indomethacin within the first 15 h of life (11). Consistently, two other studies did not confirm any significant associations between thrombocytopenia and a higher incidence of PDA/hsPDA or later PDA treatment failure (12, 13). In contrast, data from several other investigators suggest that thrombocytopenia within the 1st week of life did contribute to prolonged ductal patency (14–17) and a meta-analysis from 2015, which included 11 cohort studies involving 3,479 infants, concluded that low platelet counts within the 1st day(s) of life were marginally but significantly associated with PDA/hsPDA (18).

In terms of platelets and pharmacological DA closure, several authors reported that low platelet counts within the 1st day(s) of life were not predictive of later pharmacological treatment failure in infants with hsPDA (14, 15, 19, 20). However, recent studies have yielded conflicting results on whether a positive association between higher platelet counts just before or during cyclooxygenase inhibitor therapy and PDA closure rates exists. For example, while platelet counts before pharmacological therapy were not predictive of PDA treatment success rates, low platelet counts during pharmacological treatment were associated with an increased rate of treatment failure in a cohort of 471 preterm infants. In this study, only infants with hsPDA on day of life 3–5 received cyclooxygenase inhibitor treatment (hemodynamically significant if (i) a respiratory setback with a supplemental oxygen requirement >30% and/or mechanical ventilation, (ii) a LA/Ao ratio ≥1.4 in the echocardiogram and/or (iii) ductal diameter ≥2.5 mm, and/or (iv) a decreased end-diastolic flow in the anterior cerebral artery with a resistance index ≥0.85) (21). In addition, two reports on indomethacin found that higher platelet counts just before treatment initiation were associated with higher success rates (22, 23), while others were unable to demonstrate such an association (24, 25). Nonetheless, similar to the results obtained for spontaneous ductal closure (18), a recent meta-analysis (eight studies including 1,087 infants) revealed a moderate but significant association between pretreatment thrombocytopenia and failure of pharmacological PDA closure (26). Of note, cyclooxygenase inhibitors are known to adversely affect platelet plug formation in adults. The few available data from preterm infants suggest that inhibition of platelet plug formation in neonates may occur, but seem to be of limited clinical significance under cyclooxygenase inhibitor treatment (27). However, one might speculate that even moderate alterations in platelet function may become relevant in severely thrombocytopenic infants.

Taken together, the combined evidence suggests the existence of a moderate but detectable impact of platelets on both spontaneous and pharmacological PDA closure in preterm infants. However, it should be noted, that most studies were retrospective in nature, and thus, even the positive studies cannot conclude on any causality as relevant confounding factors might have been missed by the analyses. In addition, the remarkable heterogeneity in study design (selection criteria, genetic background, and timing of platelet counts), diagnostic protocols (timing of echocardiography and criteria used to define hemodynamic significance), and differences in treatment approaches (timing, dosage, and application route) may have influenced the comparability of the available studies (18, 26, 28).

A recent randomized-controlled trial on platelet transfusions addressed the clinical implications of the aforementioned associations between low platelet counts and failure of hsPDA closure in preterm infants (<35 weeks of gestational age) (29). The authors compared liberal platelet-transfusion criteria (platelet counts were maintained >100,000 /μL) vs. restrictive criteria (platelet count <20,000/μL, clinical bleed, platelet count <50,000/μL and requiring a major non-neurosurgical procedure, or platelet count <100,000/μL and requiring a neurosurgical procedure), and the impact of either approach on hsPDA closure (*n* = 22 in each group). All subjects received standard co-treatment with nonsteroidal anti-inflammatory drugs. There was no significant difference in the time required to achieve PDA closure (median of 72 h in both groups, *p* = 0.697). However, 41% of all infants in the liberal transfusion group were found to develop intraventricular hemorrhage (IVH, any grade), while IVH was only seen in 4.5% of the infants allocated to the restrictive transfusion group (*p* = 0.009) (29). Potential mechanisms that might contribute to the increased rate of IVH in liberally transfused infants include hemodynamic alterations due to volume challenge by transfusions and a possible modification of periventricular capillary function by transfused platelets.

## Platelet Indices and PDA

Considering the contradictory results on platelet counts and their possible associations with PDA incidence and treatment success, several experts have speculated that platelet indices such as mean platelet volume (MPV), platelet distribution width (PDW), or platelet mass (calculated by multiplying platelet count by MPV) may be more useful in predicting PDA and/or treatment response. Alyamac Dizdar et al. (14) reported that high PDW values within the first 3 days of life were associated with PDA. Similar to Dani et al. (15) they did not find any association between MPV and PDA incidence or treatment response. Another group reported consistently higher PDW values in infants with hsPDA within the first 24 h after birth, but did not find any association between MPV, platelet mass, or platelet counts and hsPDA, respectively (20). However, such an association between higher platelet mass and MPV and hsPDA closure has been reported by others (30). Another group from Turkey did not find any relationship between platelet counts, MPV, or platelet mass and successful hsPDA closure by ibuprofen (19). In addition to MPV, PDW, and platelet mass, other hematologic indices, such as red cell distribution width-to-platelet ratio (RPR) and platelet-to-lymphocyte-ratio (PLR) have been proposed as markers for hsPDA and treatment response (31–33).

In summary, the aforementioned studies (and recent meta-analyses) on platelet indices and PDA have revealed inconsistent or even conflicting results, which were probably, at least in part, due to differences in study designs, treatment protocols, and the definition of hsPDA (28, 34). Furthermore, hematologic indices might be affected by several conditions, such as inflammation, hypoxia and/or volume status, and thus likely represent surrogate markers of neonatal well-being rather than being specific indicators of PDA/hsPDA, e.g., higher PDW values are thought to reflect altered platelet function/activation (35).

## Platelet Function and PDA Closure in Preterm Infants

Previous studies investigating the role of thrombocytopenia in spontaneous or pharmacological ductus arteriosus closure have provided controversial results. However, several investigations have consistently identified sepsis/inflammation, lower gestational age, and feto-maternal conditions such as preeclampsia as risk factors for failure of DA closure in preterm infants. Since all these factors are associated with altered platelet function, it has been speculated that platelet function, rather than platelet number, is the key determinant of PDA closure in neonates (36, 37). A significant association between lower platelet count, higher CRP level and incidence of PDA has been demonstrated, with CRP being the only independent predictive factor for PDA in a regression model (38). These findings support the theory of a combined effect of one common cause (e.g., sepsis) on both platelet number and function and failure of DA closure. A proteomics-based study on human neonatal plasma identified six differentially expressed proteins (platelet factor 4, fibrinogen, von Willebrand factor, collagen, mannose binding lectin-associated serine protease-2, fibronectin), which were associated with PDA. Interestingly, those proteins are closely related to platelet activation and plasmatic coagulation cascades (39).

Recent observational studies have also confirmed a relationship between altered platelet function and failure of DA closure in human preterm infants. Collagen-ADP closure times > 130 s (PFA-100) are more frequently observed in infants with PDA as compared to those without PDA, and longer collagen-ADP closure times represent an independent risk factor for ductal patency (40). In addition, lower median levels of platelet-derived growth factor (PDGF) were found in those infants who later required pharmacological PDA therapy vs. those who did not require such treatment (41). The same group of authors also proposed the usage of platelet-rich plasma in preterm infants with PDA as an experimental approach for PDA treatment (42–44). Recently, other investigators found lower PDGF levels in very immature infants (22–27 weeks) with failure of spontaneous DA closure (45). In addition, Ghirardello et al. (46) assessed thromboelastography at birth in VLBW infants with PDA (*n* = 151). In their study, thromboelastography at birth did not predict persistence of PDA. However, infants who failed medical treatment exhibited signs of an enhanced fibrinolysis (46). While the exact mechanisms and the clinical consequences of these findings are yet to be examined, they further indicate a role of the neonatal coagulation system in ductal closure.

It should be noted that the hemostatic system and especially platelet function in infants differ from that of adults—a fact that needs to be taken into account when designing future studies on the impact of platelet function on neonatal diseases such as PDA (47, 48). Despite developmental “defects” in platelet function, healthy full-term infants show a more effective primary hemostasis than healthy adults in whole blood tests of primary hemostasis such as bleeding time and PFA-100 (49). This has been attributed to several factors, including high levels of large von Willebrand factor (vWF) multimers, high hematocrit, and high mean corpuscular volume (MCV) values, which compensate for the impaired platelet function in newborns (47, 49).

**Figure 1:**
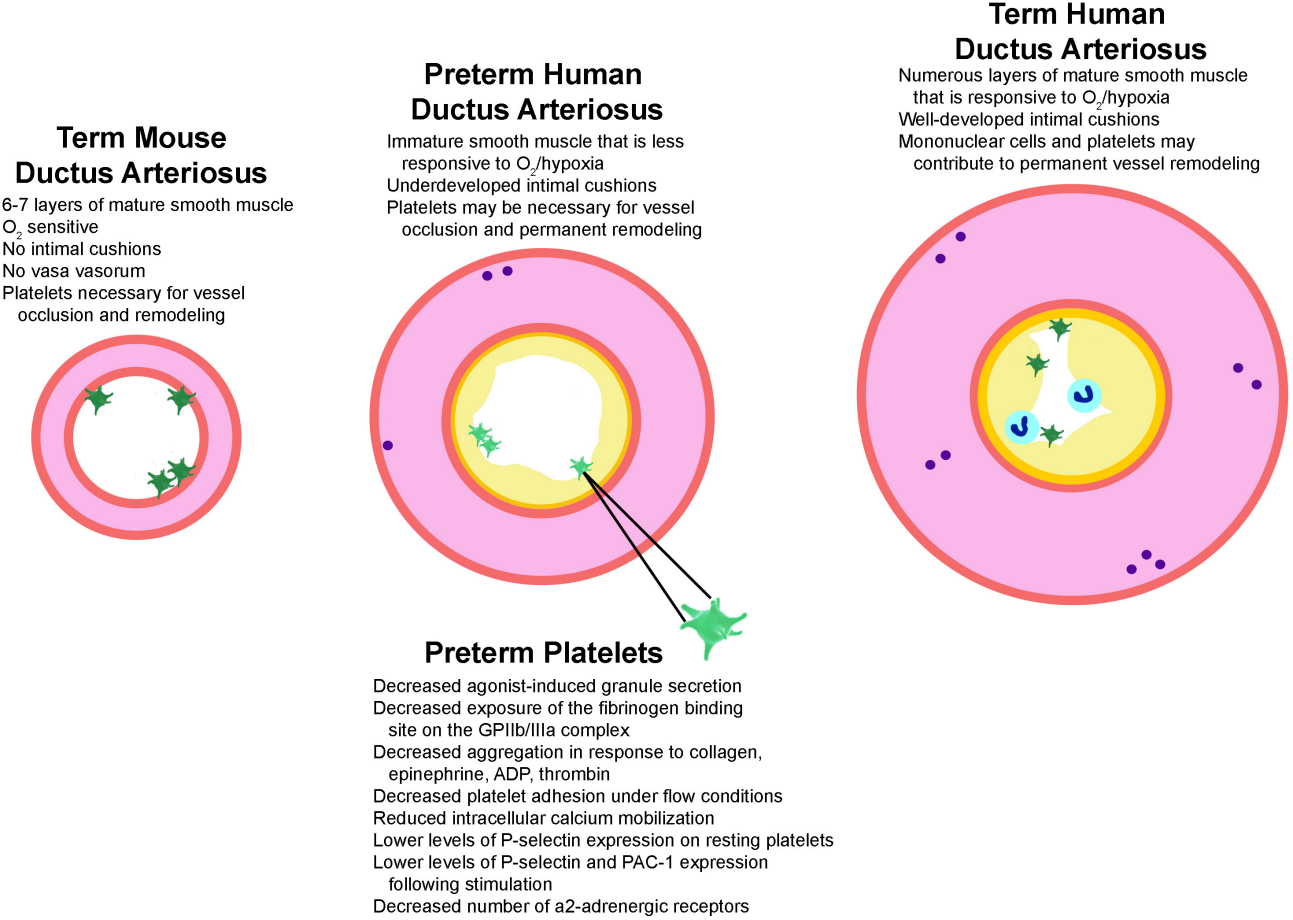
Structural factors that play a role in ductus arteriosus closure. Illustrated are the structural features known to regulate ductus closure in mice, preterm infants, and term-gestation infants. Mice have mature smooth muscle cells (pink) that constrict robustly in response to oxygen but lack intimal cushions (yellow), which may explain why platelets (green) are required for vessel occlusion and remodeling. Term-gestation infants have a thick medial layer composed of mature smooth muscle cells that are very sensitive to oxygen-induced constriction. They also have prominent vasa vasorum (purple circles) which results in local ischemic areas and hypoxic signaling during closure. A platelet plug and mononuclear cells (blue circles) adhere to the lumen during closure and promote formation of a permanent seal and remodeling. In contrast, several structural factors contribute to failed ductal closure in preterm infants. The preterm ductus has an underdeveloped media containing immature smooth muscle cells and less vasa vasorum, making it less sensitive to oxygen/hypoxia signaling. Preterm vessels also lack prominent intimal cushions and have preterm platelets. Characteristics of preterm human platelets that are associated with an enhanced vulnerability to dysfunction, which in turn might contribute to higher rates of ductal patency in immature preterm infants are also listed. ADP, adenosine di-phosphate; GPIIb/IIIa, glycoprotein IIb/IIIa; PAC-1, procaspase activating compound 1 which detects activated GPIIb/IIIa.

Platelet function may play an even larger role in closure of the preterm ductus, given that preterm vessels are often structurally underdeveloped (lack fully-formed intimal cushions) and less responsive to oxygen-induced vasoconstriction. However, in preterm infants below 30 weeks of gestational age platelet function is further impaired when compared to full-term infants (Figure 1). For example, preterm platelets show a decreased number of α2-adrenergic receptors, decreased calcium mobilization, and impaired signal transduction causing a decreased agonist-induced granule secretion and exposure of the fibrinogen binding site on the GPIIb/IIIa complex (49). More immature preterm infants also exhibit lower levels of P-selectin expression on resting platelets (50) and lower levels of P-selectin and PAC-1 following stimulation with platelet agonists (49). They also show decreased platelet adhesion and aggregation in response to platelet agonists such as collagen, epinephrine, ADP, and thrombin and longer bleeding and closure times by PFA-100 (49, 51, 52). Similar results on the developmental maturation of platelet function have recently been reported in murine fetuses. Interestingly, these investigators reported rapid platelet aggregate formation when adult platelets where transfused into the fetal circulation. However, in line with the later prospective trial by Kumar et al. (29), a retrospective analysis on the effect of platelet transfusions on PDA closure rates in preterm infants failed to demonstrate any influence of transfusions on ductal closure (53).

In addition to developmental differences in platelet function between preterm infants, term infants and adults, it is known that newly released platelets (immature platelets) differ in their functional properties as compared to older, more mature platelets (54). It has recently been shown that a lower number of mature platelets during the latter half of the 1st week of life was associated with PDA in preterm infants, while immature platelets were not independently associated with PDA (55). Importantly, a mathematical model of platelet turnover in preterm infants demonstrated significantly shorter population survival values for immature platelets and shorter platelet life spans in thrombocytopenic vs. non-thrombocytopenic neonates, indicating a preferential depletion of older, more mature platelets in thrombocytopenic infants (56).

Of note, the interaction of platelets with other circulating and tissue resident blood cells has been studied in common cardiovascular diseases such as atherosclerosis, but not in human PDA. For example, platelet–neutrophil complexes (PNCs) are increased in patients with acute myocardial infarction, in association with increased levels of neuronal guidance protein semaphorin 7A (57). Mononuclear cells contribute to ductal closure (58, 59), but their direct or paracrine interaction with platelets and other circulating blood cells in human PDA is still unclear. Likewise, red blood cells affect bleeding and thrombosis, and interact with cellular and molecular components of the hemostatic system; however, platelet-erythrocyte interactions have not been investigated in preterm infants with PDA (60).

## Conclusions

The identification of platelets as contributors to ductal closure in neonates represents a conceptual breakthrough in developmental vascular medicine. Developmentally regulated platelet-endothelial interactions contribute to normal cardiovascular transition from fetal to postnatal life (6, 7, 36). In addition, thrombocytopenia and impaired platelet function seem to impact spontaneous and pharmacological PDA closure in preterm infants, linking platelets to a specific neonatal cardiovascular disease. While the clinical implications of these findings are still controversial, during the last decade, they have stipulated further research efforts on the specific developmental differences between neonatal and adult megakaryopoiesis, platelet formation and function, and their respective roles in neonatal health and disease (61). For example, the role of platelets in retinopathy of prematurity has been recently elucidated by several investigators (62–64).

In light of the recently observed practice changes toward a less frequent and less proactive treatment approach to managing preterm PDA, clinicians still face the difficult questions on how to decide which infants will benefit from PDA closure; and on when and how PDA closure should be attempted or facilitated [(1, 2, 4)—please also refer to other articles in this special issue]. Despite the experimental and epidemiologic evidence for an association between platelets and ductal closure, current clinical evidence strongly suggests, that platelet transfusions for facilitating PDA closure in preterm thrombocytopenic infants cause more harm than good (29, 65).

Thus, further research is required to elucidate the complex interplay between PDA, shunt, circulating blood cells, including platelets, and adverse outcomes of PDA in order to define accurate biomarkers and the optimal therapeutic strategies for this still elusive condition in the most vulnerable population of preterm infants (1, 66).

## Author Contributions

HS, NT, SW, and ES drafted the manuscript and prepared display items. All other authors reviewed and critically revised the manuscript. All authors contributed to the article and approved the final version as submitted.

## Conflict of Interest

The authors declare that the research was conducted in the absence of any commercial or financial relationships that could be construed as a potential conflict of interest.
